# Supplementary material for: The carbonyl-lock mechanism underlying non-aromatic fluorescence in biological matter
Source: Nat Commun. 2023 Nov 13;14:7325. doi: 10.1038/s41467-023-42874-3 (PMC10643446; doi:10.1038/s41467-023-42874-3)
Supplement: Supplementary file 1 — Supplementary Information [file 41467_2023_42874_MOESM1_ESM.pdf]

Supplementary Information for The Carbonyl-Lock Mechanism Underlying Non-Aromatic Fluorescence in Biological Matter

Gonzalo Díaz Mirón[1], Jonathan A. Semelak[1], Luca Grisanti[2], Alex Rodriguez[3], Irene Conti[4], Martina Stella[3], Jayaramakrishnan Velusamy[5], Nicola Seriani[3], Nada Došlić[2], Ivan Rivalta[4,7], Marco Garavelli[4], Dario A. Estrin[1], Gabriele S. Kaminski Schierle[6], Mariano C. González Lebrero[1], Ali Hassanali[3,*], and Uriel N. Morzan[3,*]

[1]Departamento de Química Inorgánica, Analítica y Química Física, Instituto de Química Física de los Materiales, Medio Ambiente y Energía (INQUIMAE), Facultad de Ciencias Exactas y Naturales, Universidad de Buenos Aires, C1428EHA Buenos Aires, Argentina.
[2]Division of Theoretical Physics, Ruder Bošković Institute, 10000 Zagreb, Croatia
[3]Condensed Matter and Statistical Physics, The Abdus Salam International Centre for Theoretical Physics, 34151 Trieste, Italy
[4]Dipartimento di Chimica industriale "Toso Montanari", Università di Bologna, Viale del Risorgimento 4, 40136 Bologna, Italy
[5]Division of Physical Chemistry, Ruder Bošković Institute, 10000 Zagreb, Croatia
[6]Chemical Engineering and Biotechnology, University of Cambridge, CambridgeCB3 0AS, United Kingdom.
[7]ENSL, CNRS, Laboratoire de Chimie UMR 5182, 46 allée d'Italie, 69364 Lyon France
[*]ahassana@ictp.it umorzan@ictp.it

The left panel of Supplementary Figure 1 shows that during the 250 fs after excitation L-glu has almost completely decayed to the ground state (99% of the trajectories relax to the S_0 state in this period. The non-radiative relaxation to the ground state occurs between 60-150 fs. This extremely short lifetime is explained by the passage trough an S_1-S_0 CoIn. Conversely, L-pyro(amm) remains in the S_1 state with an almost negligible population transfer to the ground state (only 2% of the trajectories relax to the

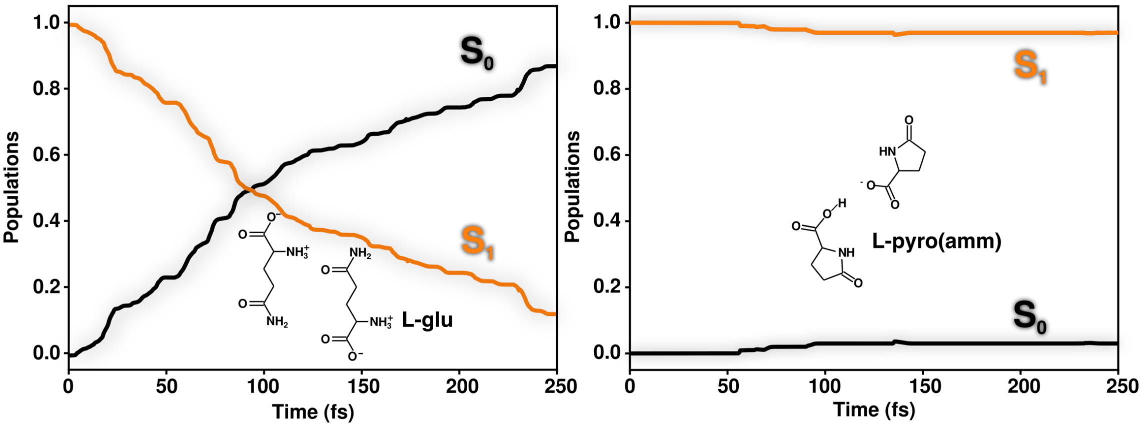

Supplementary Figure 1: Average populations of S_1 (orange) and S_0 (black) electronic states for the L-glu (left) and L-pyro(amm) (right) systems.

Supplementary Figure 2 shows the distribution of the AIMD projections in the $S_1 \rightarrow S_0$ relaxation pathway, as well as the time evolution of these projections. The observed distribution is bimodal with one maximum corresponding to the S_0 state and the other to the S_1 state.

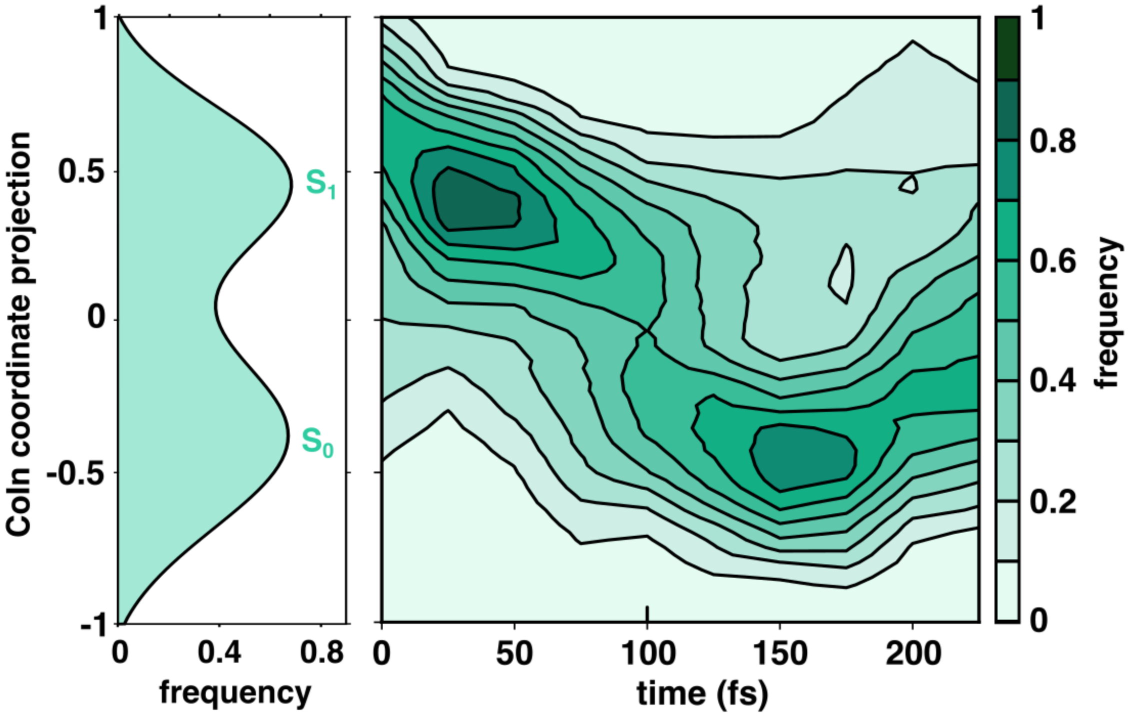

Supplementary Figure 2: Relaxation pathway histogram. The left panel represents the overall histogram of the AIMD projection in the relaxation pathway vector, discriminating the components associated with S_0 (black), S_1 (orange), and CoIn (green) dynamics. The right panel shows the time evolution of the relaxation pathway projected AIMD histogram.

Supplementary Figure 3 shows the displacements along the amide deplanarization degree of freedom during the NAMD simulations of L-glu. The mode was described computing the carbonyl C distance to the plane formed by N, O and C neighboring atoms. In the S_1 state the amide plane is strongly distorted, and the decay to the ground state is accompanied by a re-planarization.

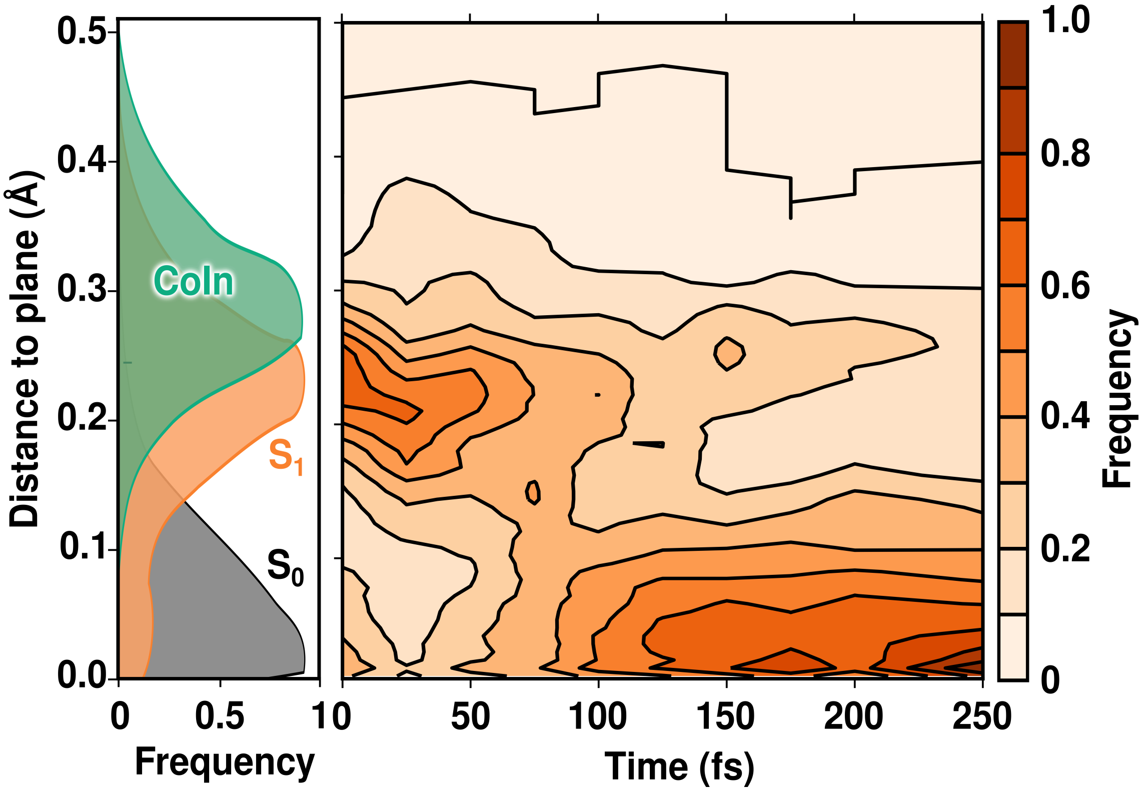

Supplementary Figure 3: Distance of the C atom of the carbonyl to the plane formed by the atoms neighbors in the amide group of L-glu. Top panel show the evolution time in all the NAMDs and lower panel show the planarization as a histogram in the different electronic states as well as in the CoIn. (The histograms were normalized independently.)

L-glu system exhibits a deplanarization of the amide group in the S_1 state as a consequence of the stretching of the carbonyl group. Upon stretching, the carbonyl looses its double bond character breaking the characteristic planar arrangement of its neighbors. In the CoIn, L-glu increases its deplanarization, which is consistent with the observations made by Grisanti et al[1], showing that the deplanarization minimizes the energy gap during the excited state dynamics of several amyloid model systems.

Supplementary Figure 4 depicts the HOMO and LUMO orbitals of L-glu and L-pyro(amm). The transition between these two is the primary component of the S_1 state. In both L-glu and L-pyro(amm) the S_1 state corresponds to a charge transfer transition between two hydrogen-bound molecules.

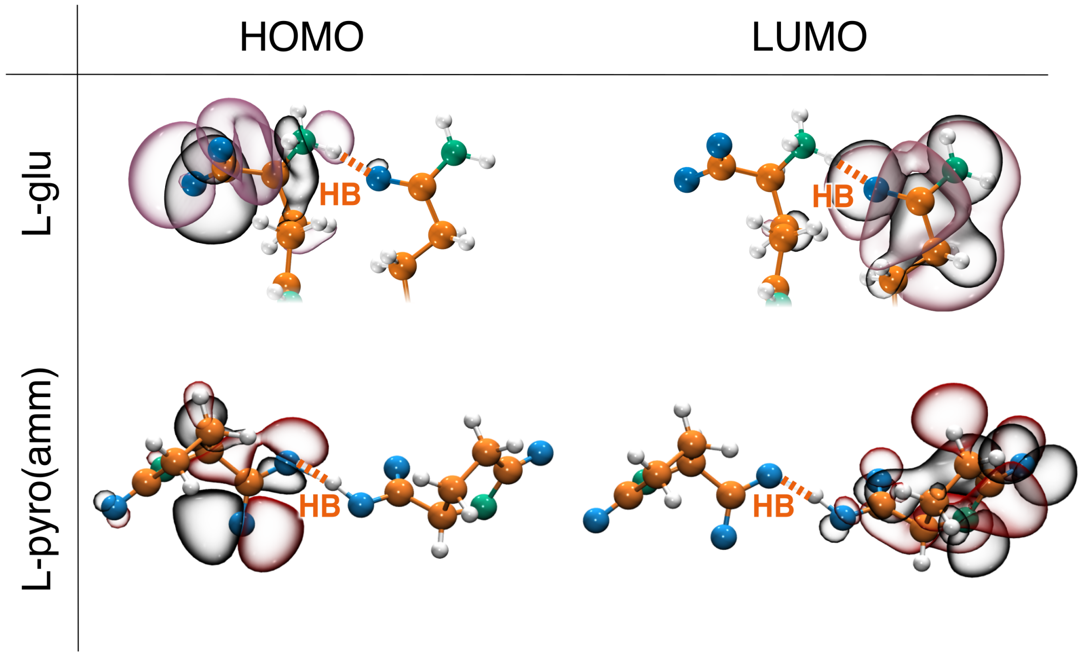

Supplementary Figure 4: HOMO and LUMO orbital representation of L-glu and L-pyro(amm).

Supplementary Figure 5 shows a comparison between the average CoIn structures obtained from TDDFT-based NAMD simulations and CASPT2 CoIn optimizations (see methods section). The resemblance between the two levels of theory serves as a validation for the TDDFT description near the S_1-S_0 CoIn.

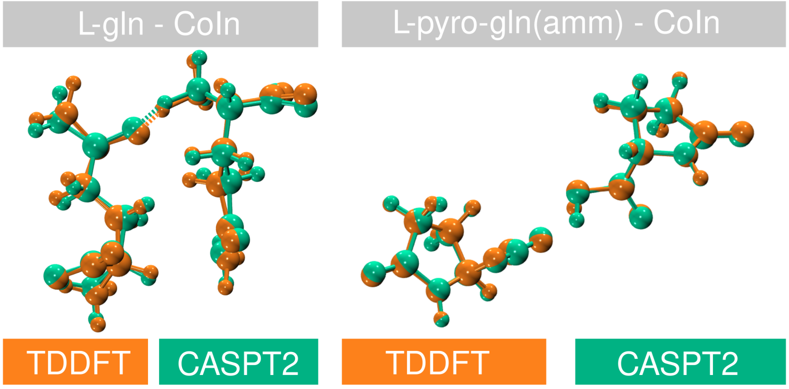

System	RMSD [Å]
L-glu Vacuum	1.70
L-glu QM/MM CI1	0.83
L-glu QM/MM CI2	0.75
L-pyro(amm) Vacuum	0.10
L-pyro(amm) QM/MM CI1	0.79
L-pyro(amm) QM/MM CI2	0.66

Supplementary Figure 5: Validation of our TDDFT NAMD simulations in the vicinity of the CoIns. The left panel shows the structural comparison between the average CoIn geometry visited by L-glu in our TDDFT-based NAMD simulations (orange plot), and our CASPT2 CoIn optimizations. The right panel shows an analogous comparison for the case of L-pyro(amm).

Supplementary Figure 6 shows the S_1-S_0 energy gap as a function of time for a selected trajectory. The black curve corresponds to a freely evolving system decaying to the ground state at ≈160 fs, and the orange curve corresponds to the same trajectory with an harmonic constraint on the CN DoF. The latter is in contact but not directly involved with the $S_0 \rightarrow S_1$ relaxation pathway DoFs. As a consequence, the applied constraint does not delay significantly the relaxation, and the system decays to the ground state only ≈30fs later than the unconstrained trajectory.

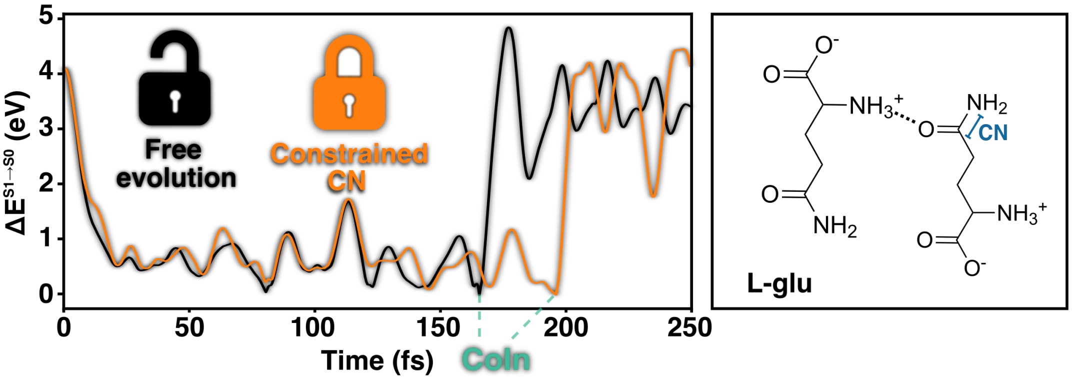

Supplementary Figure 6: Energy gap for a selected L-glu trajectory evolving freely (black curve) or applying an armonic constraint (orange curve) on the CN distance. Both trajectories reach the CoIn with a difference of ≈30fs.

Supplementary Figure 7 shows the interplay between the different degrees of freedom (DoFs) involved in the $S_1 \rightarrow S_0$ relaxation pathway of L-glu. Each plot illustrates the displacement of a given DoF during a NAMD trajectory. The figure reveals the crossed influence between the different DoFs, i.e. the extent by which a constraint in a given DoF affects the displacement on another DoF. This data is employed to build the bar chart in Figure 2 in the main text.

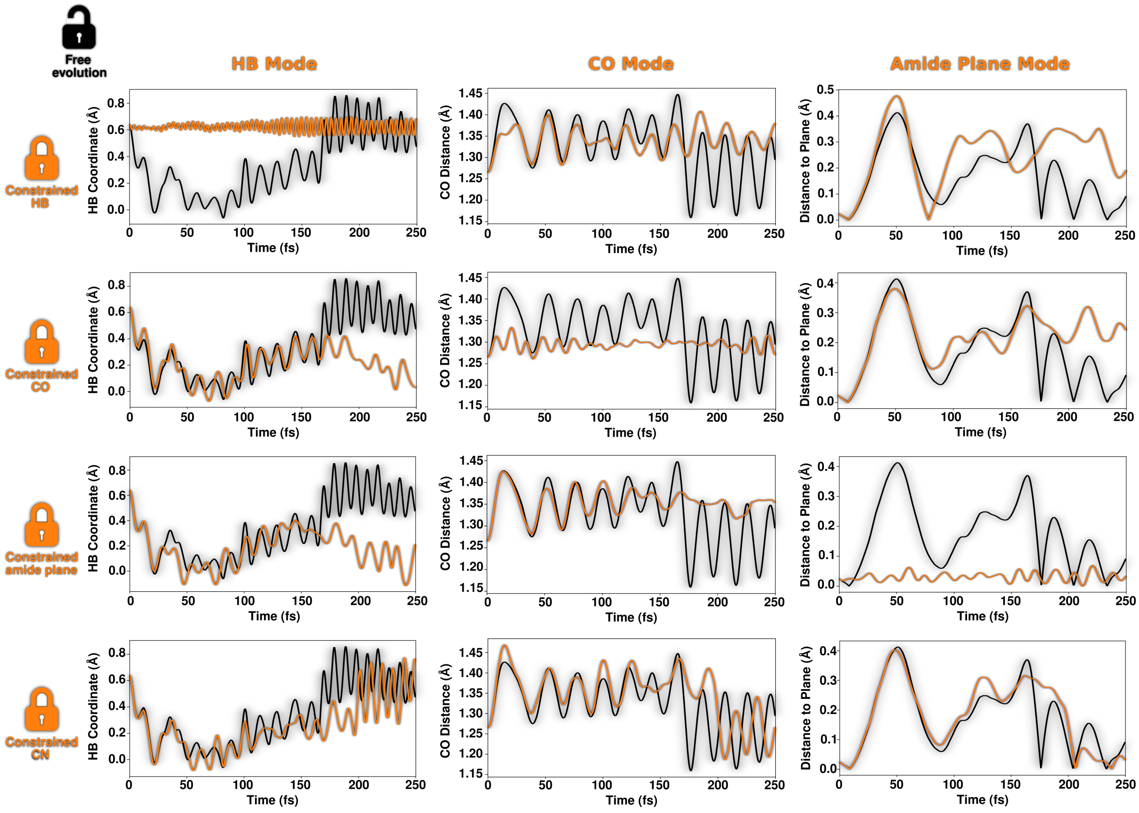

Supplementary Figure 7: Temporal evolution for the degrees of freedom integrating the $S_1 \rightarrow S_0$ relaxation pathway in L-glu trajectory evolving freely (black lines) or under an harmonic constraint applied on selected degrees of freedom. Left panels show the evolution of the HB coordinate. Mid panels show the evolution of the CO distance. Right panels show the evolution the amide planarization mode. The orange curves in the first (top) row plots illustrate the time evolution of the corresponding degrees of freedom under an applied harmonic constraint in the HB mode. The orange curves in the second row are the result of applying a constraint in the CO distance. In the third row the applied constraint is on the amide plane, and in the forth row the applied constraint is on the CN distance.

Supplementary Figure 8 shows the distribution of instantaneous radiative $S_1 \rightarrow S_0$ transition probabilities, along the transient dynamics in the S_1 state of L-glu (left panel) and L-pyro(amm) (right panel). The instantaneous radiative decay probabilities are determined employing the oscillator strength of the vertical transitions at every nuclear configuration sampled in the MD simulations. Both distributions of L-glu and L-pyro(amm) show a similar order of magnitude in the oscillator strength, indicating that the origin of fluorescence in L-pyro(amm) is primarily due to the S_1 lifetime increase rather than an enhancement of the instantaneous radiative decay probability.

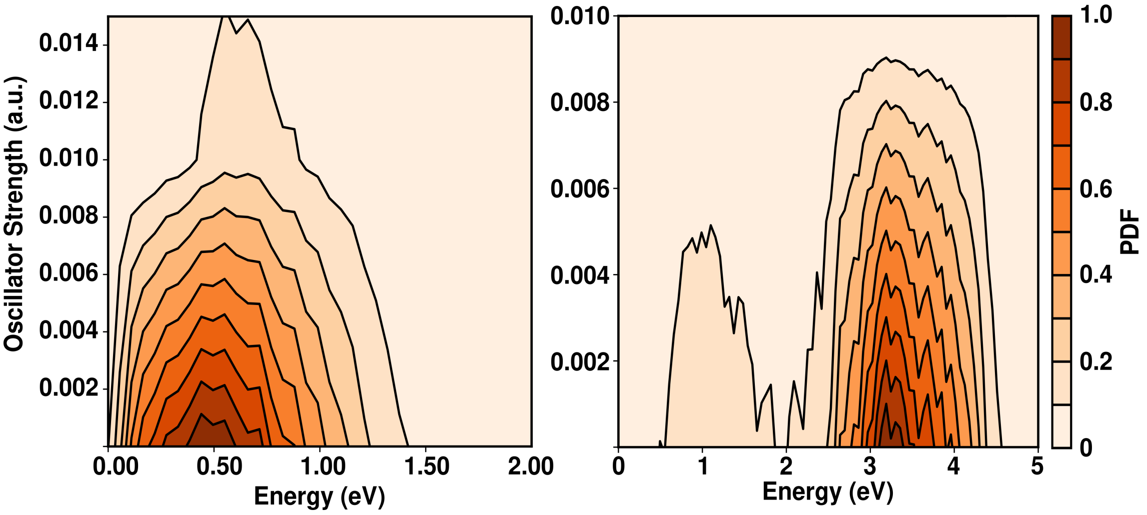

Supplementary Figure 8: Oscillator strength distribution associated to the radiative $S_1 \rightarrow S_0$ transition for L-glu (left panel) and L-pyro(amm) (right panel).

Supplementary Figure 9 illustrates the crystal structures of the L-glu and L-pyro(amm) obtained by Stephens et al, [2] as well as the dimer model systems employed throughout this work. In order to preserve the molecular arrangement of the crystal structure we applied soft harmonic constraining potentials (a force constant of 100 Kcal/molÅ) to the atoms indicated in Figure 9.

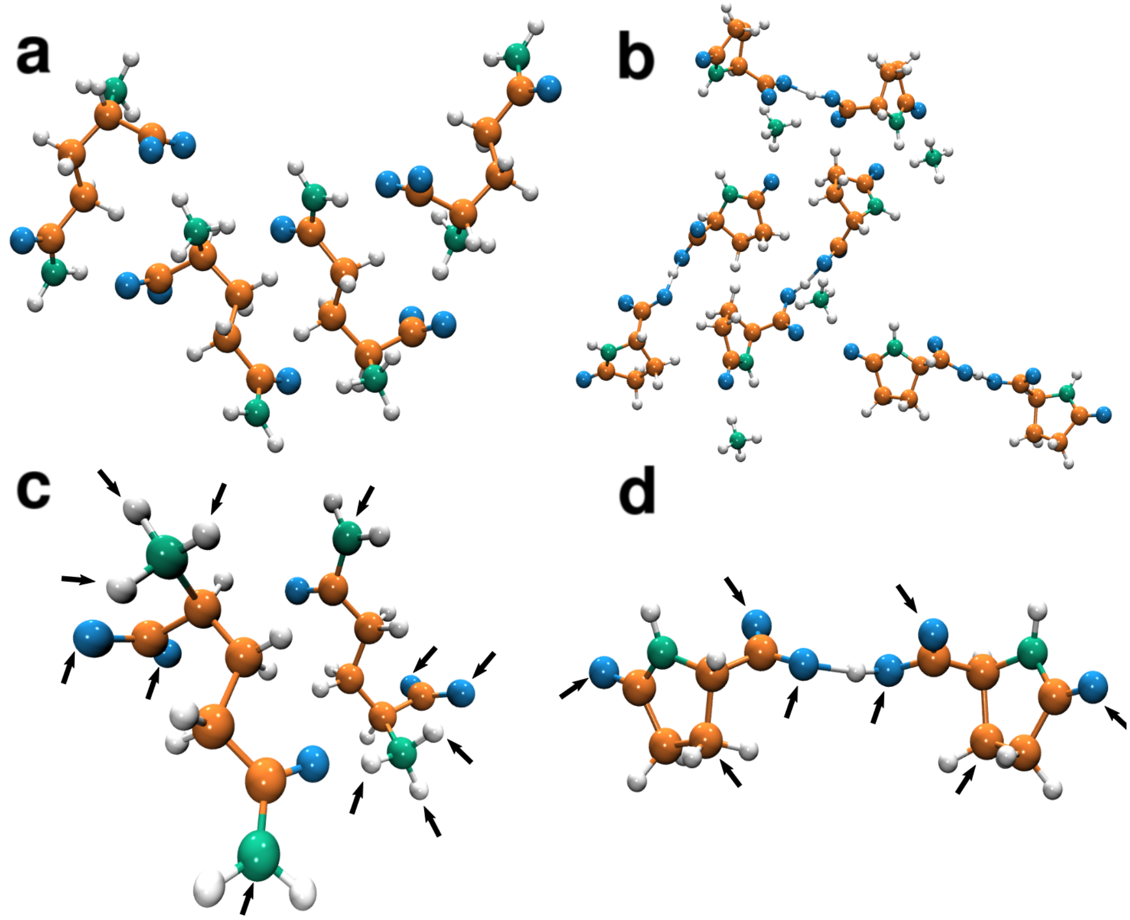

Supplementary Figure 9: Molecular structures. a) L-glutamine, b) L-pyroglutamine-ammonioum, c) Model system of L-glu, d) system model for L-Pyro-amm, named L-pyro(amm) since it does not contain the ammonium ion explicitly but it preserves the structural arrangement of the system in presence if ammonium. The black arrows on system models (panel c and d) represent the positions where the soft constrains were applied.

Supplementary Figure 10 shows the influence of the α parameter selection in the relative statistical weight of the different geometric fluctuations during the NAMD trajectory of L-glu. When $\alpha \to \infty$ the Arrhenius term in equation 7 in the main text is equal to 1, and all the configurations explored during the NAMD trajectories are equally weighted in the relaxation coordinate. As discussed in the methods section, this introduces elements to the total variance that are not necesarily related

to the decay process itself. In contrast, when $\alpha = 1$ only configurations that are in the vicinity of the CoIn have significant contribution to the overall coordinate. In this case the fluctuations that lead to the CoIn proximity are neglected in the final relaxation coordinate. With $\alpha = 100$ the energy fluctuations that occur near the S_1–S_0 CoIn are considered in the relaxation coordinate estimation.

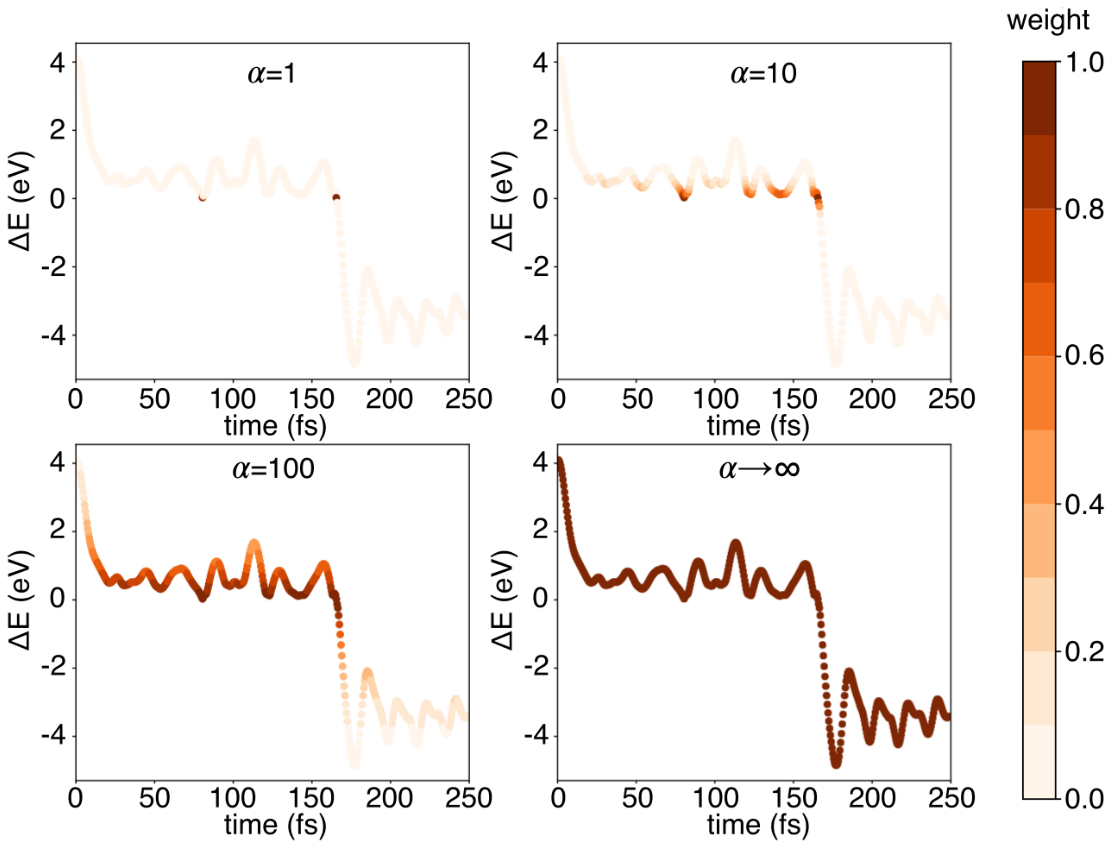

Supplementary Figure 10: Variation of the α parameter in the $S_1 \rightarrow S_0$ relaxation coordinate for a selected NAMD trajectory of L-glu. The vertical axis in the four panels show the energy difference between the S_1 and S_0 states, and the horizontal is the time evolution axis. The dot colors represent the normalized value of the Arrhenius term in expression 7 in the main text, which can be interpreted as a statistical weight of the corresponding geometry fluctuation in the relaxation coordinate.

Supplementary Figure 11 shows the simulated fluorescent spectrum of L-Pyro(amm) model, in reasonable agreement with the experimental result (the simulated maximum is only shifted ≈ 30 nm/0.2 eV from the experimental one).

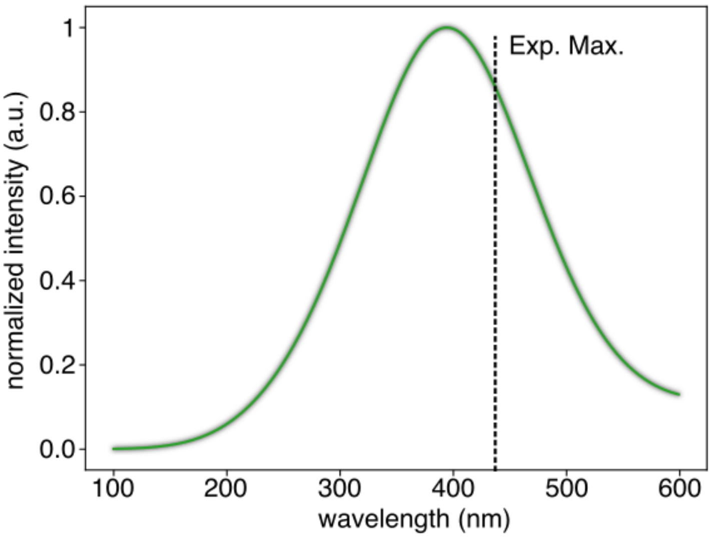

Supplementary Figure 11: Fluorescence spectrum of the L-pyro(amm) isolated dimer model system.

Supplementary Figure 12 shows the different time-dependent excited state populations of the small model systems, inspired in the amyloid sequence Aβ_{30-35} [3].

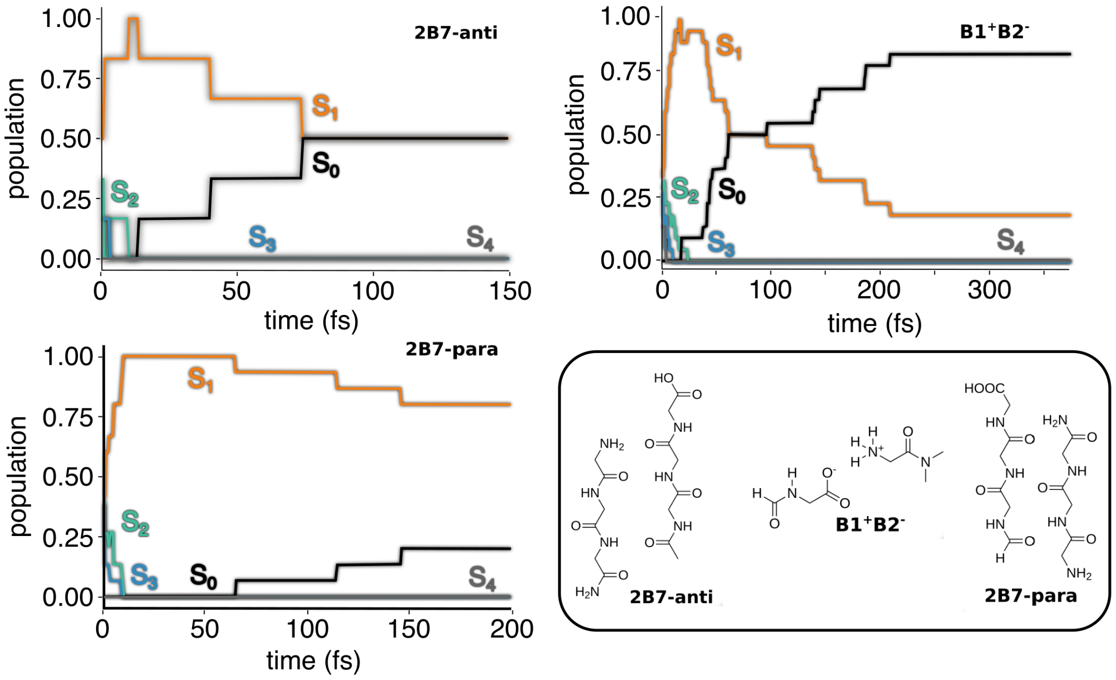

Supplementary Figure 12: Average populations of S_1 (orange) and S_0 (black), S_2 (green), S_3 (blue), S_4 (gray) electronic states for the 2B7-anti (left panel), 2B7-para (middle panel), and $B1^+B2^-$ systems.

We performed QM/MM non-adiabatic dynamics simulations comparing two limiting cases: (a) when the ammonium is included in the MM region, and (b) when the ammonium ion is included in the QM region (see Figure 13). In both cases we observe a marginal decay to the ground state, in agreement also with the isolated dimer models. Furthermore, no significant change in the relevant excited state modes is observed. We have now included this important discussion in the new version of the manuscript.

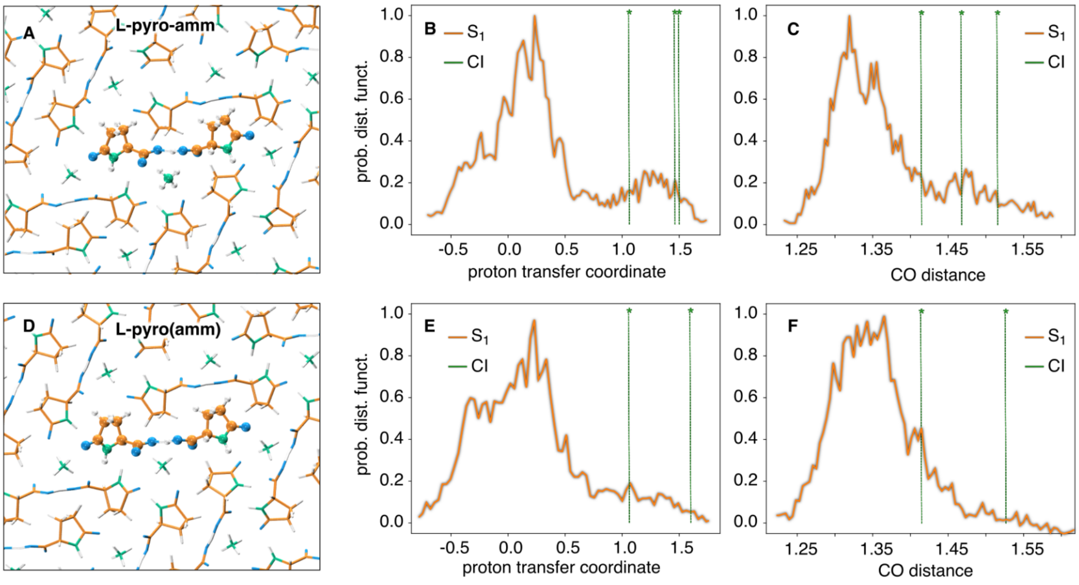

Supplementary Figure 13: Influence of ammonium ion in the non radiative decay of L-pyro system. The QM/MM models for L-pyro-amm and L-pyro(amm) are shown in panel A and D, respectively. The QM region is shown with a balls and sticks representation while the MM region is depicted only with sticks. The difference between the two models is that in L-pyro-amm the ammonium ion is included in the QM region, while in L-pyro(amm) it is included in the MM region. Panels B and E show the excited state distribution of the proton transfer coordinate for L-pyro-amm and L-pyro(amm), respectively. Panels C and F show the excited state distribution of the CO distance, defined as $d_{\text{O-H}} - d_{\text{O'-H}}$ (where O and O' identify the two carboxyl oxygens involved in the HB) for L-pyro-amm and L-pyro(amm), respectively.

Supplementary Figure 14 depicts the active space employed for the CASPT2 calculations, both at the full-QM or QM/MM level.

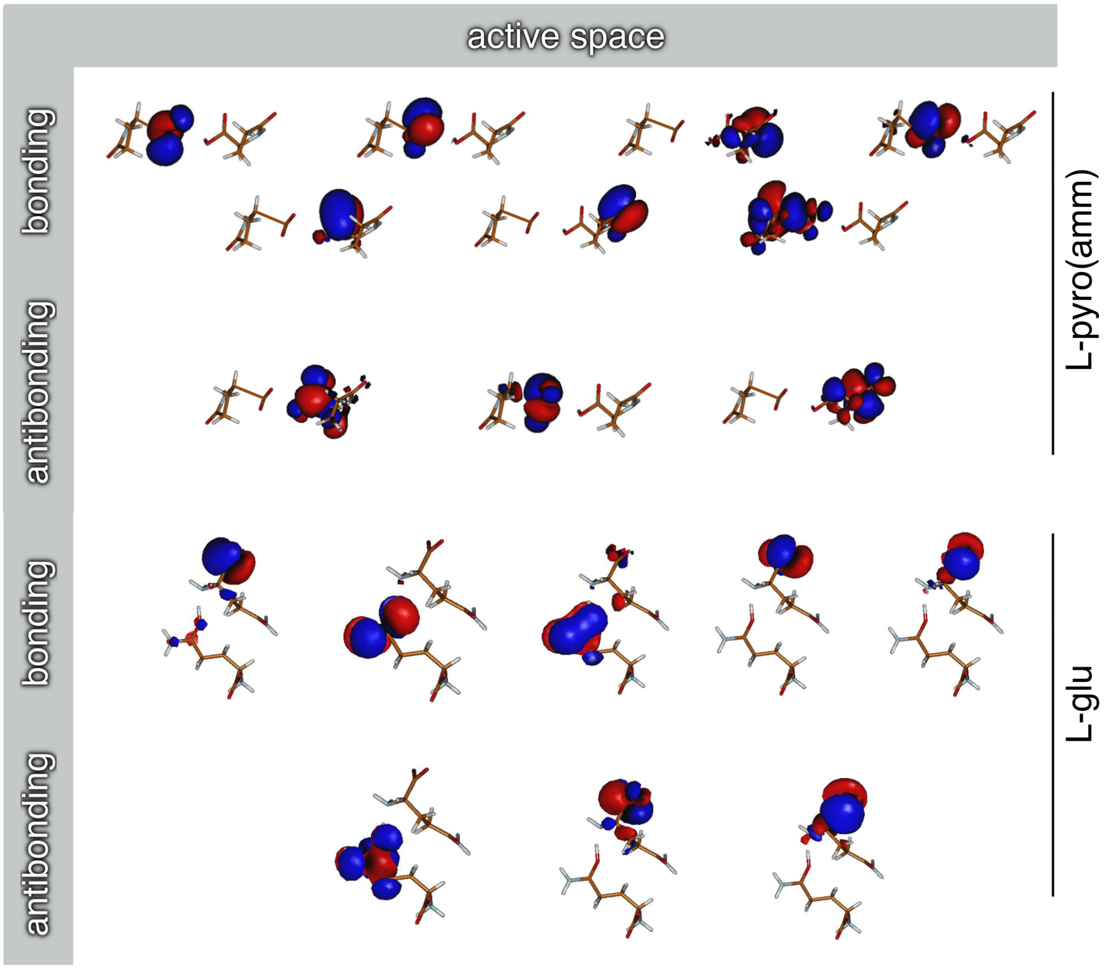

Supplementary Figure 14: Active space employed for our CASPT2 calculations.

Supplementary References

[1] Grisanti, L.; Sapunar, M.; Hassanali, A.; Došlić, N. Toward Understanding Optical Properties of Amyloids: A Reaction Path and Nonadiabatic Dynamics Study. *Journal of the American Chemical Society* **2020**, *142*, 18042–18049, PMID: 32966740

[2] Stephens, A. D. et al. Short hydrogen bonds enhance nonaromatic protein-related fluorescence. *Proceedings of the National Academy of Sciences* **2021**, *118*, e2020389118

[3] Pinotsi, D. et al. Proton Transfer and Structure-Specific Fluorescence in Hydrogen Bond-Rich Protein Structures. *Journal of the American Chemical Society.* **2016**, *138*, *9*, *3046-3057*
